# The *Terminalia laxiflora* modulates the neurotoxicity induced by fipronil in male albino rats

**DOI:** 10.1042/BSR20181363

**Published:** 2019-03-01

**Authors:** A.A. Khalaf, Mona K. Galal, Marwa A. Ibrahim, A.A. Abd Allah, Mostafa M. Afify, Rasha Refaat

**Affiliations:** 1 Department of Forensic Medicine and Toxicology, Faculty of Veterinary Medicine, Cairo University, Giza, Egypt; 2 Department of Biochemistry and Chemistry of Nutrition, Faculty of Veterinary Medicine, Cairo University, Giza, Egypt; 3 Central Lab of Pesticides, Dokki, Giza, Egypt; 4 Forensic Medicine and Clinical Toxicology Department, Faculty of Medicine, Beni-Suef University, P.O. 62511, Beni-Suef, Egypt; 5 Department of Phytochemistry and Plant Systematics, National Research Center, Dokki, Giza, Egypt

**Keywords:** apptosis, fipronil, neurotoxicity, terminella laxiflora

## Abstract

The extensive use of fipronil (FPN) may trigger hazards to more than insects. The present investigation was carried out to evaluate the abrogating role of *Terminalia laxiflora* (TL) methanol extract (TLE) against the neurotoxic effects provoked by FPN. Fourty male albino rats were assigned into four equal groups. The first group served as control, the second one was orally administered FPN (10.5 mg/kg BW), the third group was given combination of FPN and TLE) (100 mg/kg BW), and the fourth one was orally given TLE. Our findings highlighted the efficacy of TLE as a neuroprotectant through a significant reduction in malondialdehyde (MDA) content by 25.8%, elevations of the reduced glutathione (GSH) level, catalase (CAT,) and superoxide dismutase (SOD) activities by 30.9, 41.2, and 48.2% respectively. Consequently, the relative mRNA levels of both Bax and caspase-3 were down-regulated by 40.54% and caspase-3 by 30.35% compared with the control group. Moreover, restoration of the pathological tissue injuries were detected. In conclusion, TLE proved to be a potent neuroprotective agent against the FPN-induced toxicity.

## Intorduction

Fipronil (FPN) CAS number: (120068-37-3) is the N-phenylpyrazole insecticide [1] that is extensively used to control pests in diverse cereal crops and in public health [2]. It is more effective than organophosphate, carbamate, and pyrethroids insecticides against several species of insects [3]. FPN is utilized in household applications as well as commercial agricultural and veterinary purposes [4]. It is being used to control fleas and ticks on the pets [5]. FPN is neurotoxic to insects and the primary mode of action refers to interfering with the passage of chloride ions through the γ-amino butyric acid (GABA) chloride channel of the central nervous system that causes uncontrolled hyperexcitation of insects at low doses and convulsions leading to insect death at high doses [6]. FPN is more toxic to insects (LD_50_ = 0.004 microgram/bee) than mammals [7] and has moderate acute oral toxicity LD_50_’s ranging from 40 to 100 mg/kg body weight in rats and mice [8]. Currently, exposure to phenylpyrazole pesticides are a global public health issue and concerns are increased regarding the relative safety of this pesticide because FPN have been classified as class C – possible carcinogen [9]. Besides, FPN is highly toxic to many non-target organisms, such as honeybees, crustaceans, fish, rabbits, and birds [10], in which it can bind to mammalian GABAC and GABAA receptors [11], as well as its sulphone metabolite and FPN – desulphinyl (a photodegradation product), were displayed to be more toxic to insects and non-target species than the parent compound. FPN was documented to have highly toxic effects on the liver, thyroid, and reproductive functions in non-target species [12]. Currently research concerns are directed to the plant-based medicine due to its effectiveness, few adverse effects, and low price [13,14]. Several phytochemicals have been shown to exhibit substantial protection against neurotoxicity in animal models by recovering the antioxidant status [15]. Plant flavonoids extend a multiplicity of neuroprotective actions within the brain, including a potential protection to the neurones against injury induced by neurotoxins, through promoting memory, cognitive function, and learning, besides the ability to restrain neuroinflammation [16]. The genus *Terminalia* is the second largest genus of Combretaceae family consisting of 200 species, distributed in the tropics and subtropics. Approximately 30 species of *Terminalia* are found in Africa [17]. Approximately 200 woody species of *Terminalia* are used as resources in the timber, pharmaceutical, and leather industries [18]. Several biological activities of *Terminalia* species have been reported such as antibacterial, antifungal [19], anticancer [20], anti-inflammatory [21], hypocholesterolemic [22], anti-ulcer activity [23], anticaries agent [24], antiviral [25], antioxidant, and melanin inhibitory activity [26]. *T. laxiflora* (TL) is a common indigenous tree in the woodland and semi-arid savannah of Sudan with high multipurpose potentials [27]. The plant has a variety of medicinal applications. The root bark of TL is used traditionally as a gastric stimulant to prevent and cure diarrhea in infants and children, aids digestion, and relieves constipation in adults. In addition, it is used to treat wounds and strains [28]. The extensive use of FPN may pose a great threat to animals and humans, therefore, this report is aimed first, to evaluate the neurotoxic effects of subchronic exposure to FPN on the oxidant/antioxidant status and some apoptotic biomarkers of male rats. Second, to examine whether the *T. laxifolra* extract could provide a potential protection against the adverse effects of FPN.

## Methods

### Chemicals

The chemicla formula of technical-grade FPN insecticide is: C_12_H_4_Cl_2_F_6_N_4_OS, purity: 80% WG, is a product of Cros Agro. Pesticides Company, Egypt.

### Plant material

TL root bark was collected from Khartoum, Sudan, in March 2013. The sample was identified by Prof. Dr. Salwa A. Kawshty, Phytochemical and Plant Systematics Department, NRCA Voucher specimen (number 1132) and was deposited in the Herbarium of NRC (CAIRC, Cairo, Egypt). The bark was cut into chips, dried, and powdered for *in vitro* analysis.

### Extraction and isolation

Dried and powdered TL root bark (1 kg) was exhaustively extracted with 3 l of 80% aq. MeOH at room temperature. The extract was concentrated under reduced pressure giving a residue (48 g). A portion of the extract (25 g) was subjected to a polyamide (CC), starting with water as eluent then decreasing the polarity by increasing the concentration of methanol up to 100%. For isolation and purification of the compounds, the elution techniques on Whatman No. 1 and 3 MM were carried out using H_2_O, 15% AcOH, and BAW (n-BuOH:AcOH:H_2_O, 4:1:5, upper layer) as eluents. Compounds were then subjected to further purification on Sephadex LH-20 for spectral analysis. Phytochemical screening of the extract was done according to methods described by Jaradat et al. [29].

### Experimental animals

Forty male albino rats were purchased from Helwan Farm of Laboratory Animals, aged 12 weeks (170 ± 10 g) maintained at the Animal Care Facilities of Central Agricultural Pesticides Laboratory (CAPL) in plastic cages under controlled temperature (23 ± 2°C), 12-h light/dark cycle and relative humidity (50 ± 5%). Water and food were available *ad libitum*. Rats were adapted to the laboratory environment for 2 weeks prior to the onset of experiments. The Local Committee approved the experiments and the protocol conformed to the Guidelines of the National Institutes of Health.

### Experimental design and sample collection

Animals were divided randomly into four equal groups with ten animals each. The first group (I) served as control; hence animals were orally given olive oil. The second group (II) was orally treated with FPN only (10.5 mg/kg BW) approximately 1/10 LD_50_ [30]. The third group (III) was treated with combination of FPN (10.5 mg/kg BW) and TL methanol extract (TLE) (100 mg/kg BW) and finally the fourth group (IV) was orally treated with dissolved TLE only (100 mg/kg BW). After 45 consecutive days of treatment, all the rats were anesthetized by diethyl ether, killed using capitation and brain were immediately removed, washed, and stored for further analysis.

### Lipid peroxidation and antioxidants parameter measurements

Brain tissue specimens from different groups were weighted and homogenized in cold PBS (pH 7.4) using Teflon homogenizer. The homogenates were centrifuged at 14000×***g*** for 20 min at 4°C. The supernatant was used to measure the malondialdehyde (MDA) level [31], superoxide dismutase (SOD) activity [32], catalase (CAT) activity [33], and reduced glutathione (GSH) concentration [34].

### Quantitative real-time PCR for caspase-3 and Bax genes

Total RNA was isolated from brain tissue using RNeasy mini kit (Qiagen) according to the instructions provided. The RNA yield and purity (a ratio of ∼2.0 is generally accepted) was evaluated using NanoDrop. The cDNA synthesis was carried out using reverse transcriptase (Invitrogen) and oligo-dT following the manufacturer’s protocol. Real-time PCR was performed in a Real-Time PCR System (Applied Biosystems, U.S.A.) using the following primers for caspase-3, forward ACTGGACTGTGGCATTGAGA, reverse AATTTCGCCAGGAATAGTAAC; for Bax gene [35], forward ACTGGACTGTGGCATTGAGA, reverse AATTTCGCCAGGAATAGTAACC. The cDNA was amplified by 40 cycles of denaturation at 95°C for 45 s, annealing at 59°C Bax and 60°C for caspase-3 for 45 s, and extension at 72°C for 45 s. During the first cycle, the 95°C step was extended to 5 min. The size of all amplicons was confirmed by 2% agarose gel electrophoresis stained with SYBR Safe DNA gel stain (Invitrogen). The *β-actin* gene was amplified in the same reaction to serve as the internal control. The assay was repeated three times, and the values were used to calculate the gene/β-actin ratio, with a value of 1.0 used as the control (calibrator) [36].

### Histopathological examination

The total brain tissue from the different groups were fixed in 10% neutral buffer formalin then brought about to obtain 4-µm paraffin-embedding sections. The tissue sections were stained with Hematoxylin and Eosin (H&E) [37].

### Statistical analysis

The analytical determinations were carried out in duplicate and results are expressed as the mean ± S.E.M. Data for multiple variable comparisons were analyzed by one-way ANOVA test followed by Duncan and Dunnett’s post hoc multiple comparison tests to analyze the significant differences (*P*<0.05) between groups using SPSS version 17 package for Windows.

## Results

### Phytochemical screening of TLE

Phytochemical analysis of methanol 80% extract of TL root bark revealed that it contained *carbohydrates, tannins, flavonoids, alkaloids, triterpenes*, while phenols and steroids were absent. The aqueous TLE was purified through chromatographic methods yielding six bioactive compounds (Table 1). Their structures were elucidated by chemical and spectroscopic analysis using UV and ESI/MS, ^1^H NMR, and ^13^C NMR, and identified as isovitexin (1), iso-orientin (2), vitexin (3), orientin (4), gallic acid (5), and ellagic acid (6).

**Table 1:** Qualitative phytochemical analysis of TLE

	Phen	Flav	Carbo	Tan	Alka	Triter	Ster
Qualitative	−	+	+	+	+	+	−

Abbreviations: Alka, alkaloid; Carbo, carbohydrate; Flav, flavonoid; Phen, phenol; Ster, Steriod; Tan., tannin; Triter, Triterpenoids (+, present; −, absent).

### Oxidative stress biomarkers

To determine the protective effect of TLE against FPN-induced oxidative damage in rat brain, we evaluate the changes in the lipid peroxidation (LPO) marker (MDA level), antioxidant enzyme activates of SOD, CAT, and GSH concentrations. According to the results present in Table 2, the FPN intoxication in group II induced significant elevation in the level of MDA the potent marker of oxidative damage as well as significant reduction in enzymatic activity for SOD and CAT and depletion in GSH concentration compared with control. In comparison with FPN-intoxicated group, TLE treatment for group III significantly increases all the antioxidant parameters (GSH by 30.9%, CAT by 41.2%, and SOD by 48.2%) and significantly reduced oxidant parameter (MDA) by 25.8% and nearly restored to the normal level.

**Table 2:** Protective effect of TLE on oxidative stress biomarkers against FPN-induced neurotoxicity

	Group I	Group II	Group III	Group IV
**CAT U/g tissue**	32.06 ± 1.9^1^	16.5 ± 1.04^2^	23.3 ± 0.4^3^	24.9 ± 0.58^3,4^
**SOD U/g tissue**	6.7 ± 0.4^1^	3.3 ± 0.12^2^	4.89 ± 0.1^3^	5.6 ± 0.28^1,4^
**GSH µmol/g tissue**	37.45 ± 0.76^1^	29.07 ± 0.7^2^	38.08 ± 1.2^1,3^	32.3 ± 1.4^4^
**MDA nmol/g tissue**	23.3 ± 0.59^1^	35.88 ± 0.9^2^	26.6 ± 1.58^3^	22.6 ± 0.42^1,4^

Data are presented as mean ± S.E.M. Mean values with different superscript numbers (1–4) in the same row are significantly different at (*P*≤0.05).

### Caspase-3 and Bax genes mRNA expression

**Figure 1:**
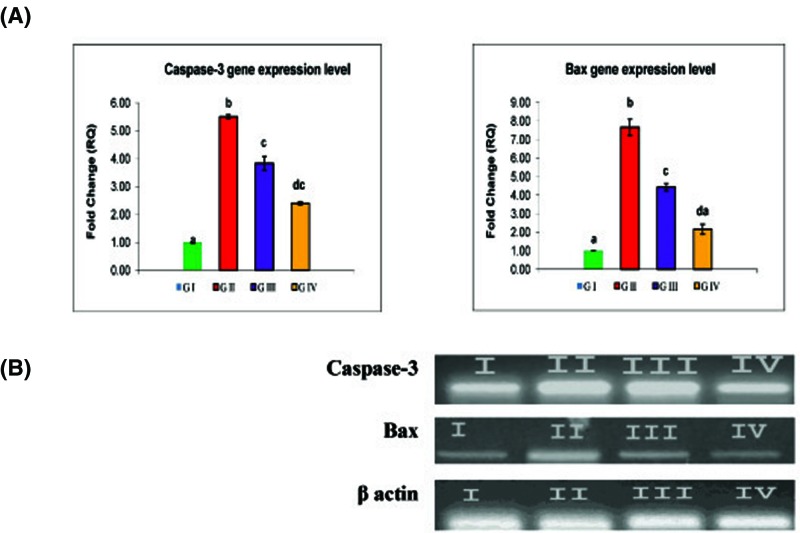
Protective effect of TLE on relative mRNA expression level of caspase-3 and Bax genes against FPN-induced neurotoxicity (**A**) Fold-change of mRNA expression of caspase-3 and Bax gene in different experimental groups using qPCR. Data are represented as mean ± S.E.M. Groups having different superscript letters (a–d) are significantly different from each other. (**B**) The results obtained by qPCR analysis were confirmed by agarose gel electrophoresis analysis of the PCR product compared with β-actin.

To evaluate whether FPN pesticide exhibited proapoptotic activity by regulating the gene expression of apoptotic pathways, we quantitated the mRNA expression of caspase-3 and Bax genes by real-time PCR (Figure 1). According to the obtained results, FPN intoxication for group II significantly up-regulated the expression level of caspase-3 and Bax genes by more than five-folds for caspase-3 gene and seven-folds for Bax gene compared with control. Gene expression analysis in the brain tissue of TLE treated rats (group III) showed a significant reduction in expression level for Bax by 40.54% and caspase-3 by 30.35% proving the anti-apoptotic effect of TLE.

### Histopathological examination

**Figure 2:**
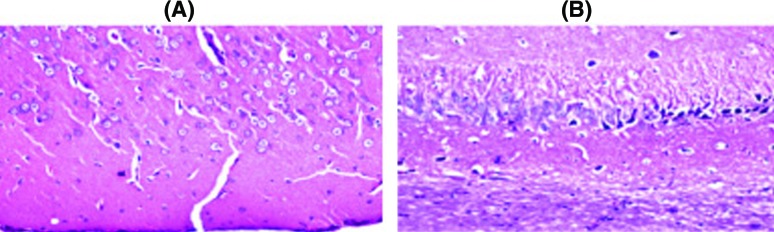
Photomicograph of neurons in control group (I) In the control group, there was no histopathological alteration and the normal histological structure of the neurones in (**A**) cerebral cortex and (**B**) subiculum in hippocampus.

**Figure 3:**
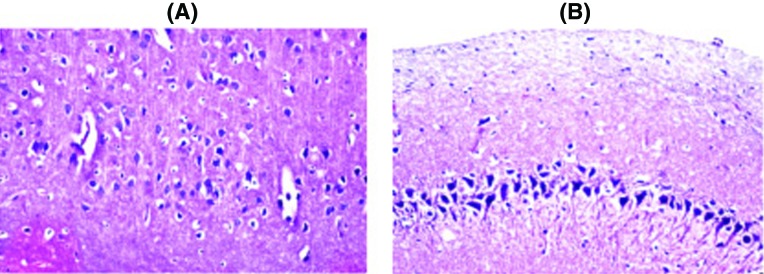
Photomicograph of neurons in FPN group (II) Nuclear pyknosis and degeneration were observed in the neurones of cerebral cortex (**A**) as well as in (**B**) subiculum of the hippocampus in FPN group.

**Figure 4:**
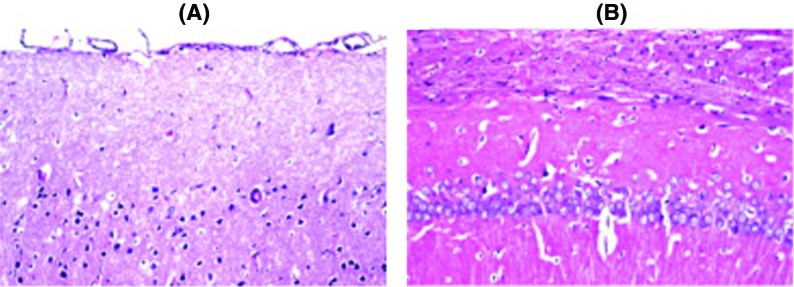
Photomicograph of neurons in TLE+FPN group (III) Nuclear pyknosis and degeneration were noticed in few neurones of the (**A**) cerebral cortex and (**B**) the subiculum in hippocampus in the *Terminelia* and FPN group.

**Figure 5:**
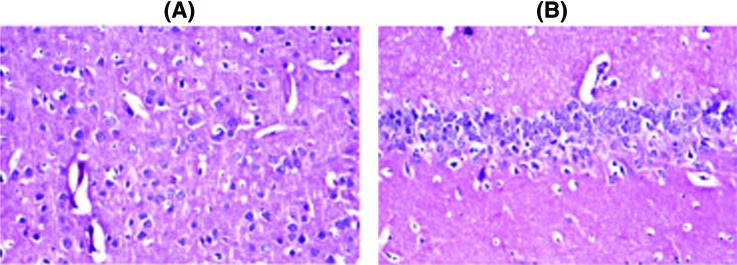
**Photomicograph of neurons in TLE group (IV).**There was no histopathological alteration in the *Terminelia* group

Histopathological examination of the brain sections in control and treated groups were illustrated in the following figures. There was no histopathological alteration and the normal histological structure of the neurones in cerebral cortex and subiculum in hippocampus were recorded in the control group (Figure 2). Nuclear pyknosis and degeneration were observed in the neurones of cerebral cortex, as well as in subiculum of the hippocampus in group II (Figure 3). Nuclear pyknosis and degeneration were noticed in few neurones of the cerebral cortex and the subiculum in hippocampus in group III (Figure 4). There was no histopathological alteration as recorded in IV (Figure 5).

## Discussion

Due to the enormous use of FPN worldwide, it is necessary to examine the toxic effects of FPN, the underlying molecular mechanisms, and search for highly effective antioxidants essential for reducing its toxicity in the future. According to the obtained data, the phytochemical analysis of methanol 80% extract of TL root bark revealed that it contained carbohydrates, tannins, flavonoids, alkaloids, and triterpenes. Chromatographic separation allowed the identification of six bioactive compounds: isovitexin, iso-orientin, vitexin, orientin, gallic acid, and ellagic acid. The structure elucidation of the pure isolated compounds were achieved by comparing the obtained data (UV, ^1^H-NMR, ^13^C-NMR, and MS) with that available in the current literature [38]. To understand the antioxidant effects of TLE against the FPN-induced oxidative damage in the brain tissue, we quantitated the MDA content, GSH level, and SOD and CAT activities. LPO is one of the major consequences of free radical-mediated injury to the brain. LPO can be used as an index for measuring the damage that occurs in membranes as a result of free radical generation [39]. MDA is a major end product of LPO, is often thought to reflect the intensity of cellular injury within the organism exposed to environmental contaminants [40]. In the present study, FPN intoxication induced significant elevation in MDA level, significant decreases in SOD and CAT enzyme activities and depleted the GSH content in brain tissue. FPN-induced oxidative damage is considered as one of the molecular mechanisms involved in pesticides-induced toxicity [41,42]. The depletion of endogenous antioxidants in the brain of rats exposed to FPN could be due to excess production of O_2_^•−^ which rapidly converted into H_2_O_2_ by SOD and to water by CAT and glutathione peroxidase (GPx) [39]. FPN can be responsible for increases in the production of ROS in cells, which lead to increased LPO of the cell membrane and subsequent cellular damage reflected by excessive accumulation of MDA [42]. Searching for agents providing protection against LPO and enhancing antioxidant enzyme defense system is a rational approach for therapy of several neurodegenerative diseases [43]. TLE treatment in group III reverted the oxidative damage induced by FPN through significant reduction in MDA level and elevation antioxidant biomarkers suggesting that TLE acted as a potent antioxidant against oxidative stress induced by FPN. Strong antioxidant and radical scavenging activity of TLE is mostly related to the hydroxyl group in gallic acid and ellagic acid [44] which are well-known natural antioxidants [45]. Plant polyphenols present in the extract are multifunctional which can act as reducing agents, hydrogen atom donors, and singlet oxygen scavengers [46]. Ellagic acid exhibited neuroprotective effects against oxidative damage in PC12 cell [47]. Tannins and flavonoids are well known to have antioxidant properties and anti-inflammatory activity [48–49] and may be responsible for significant rise in the endogenous antioxidants of treatment groups in the present study. Arjunolic acid (triterpene) has been shown to prevent the decrease in levels of SOD, CAT, GSH, and ceruloplasmin ascorbic acid and provided significant protection against LPO [50]. Oxidative stress has been suggested as one of the major risk factors exacerbating neuronal loss [13,51]. One of the outstanding characters of neurodegenerative diseases is aberrant neuronal death [52]. To determine whether FPN induced the neuronal cell death, we further tested the apoptotic role of FPN in neuronal tissue through determination of mRNA expression level of caspase-3 and Bax gene. According to the data obtained in Figure 1, FPN intoxication induced significant elevation in *caspase-3* and *Bax* mRNA expression level. Activation of caspases is often used as a marker of apoptosis. Caspases are a family of enzymes crucial for initiating and executing apoptosis. In addition, apoptosis is regulated by Bcl-2 family proteins, including anti-apoptotic proteins such as Bcl-2, and pro-apoptotic proteins such as Bax [53]. When the proapoptotic Bax protein is overexpressed in cells, apoptotic death in response to death signals is accelerated [54]. Oxidative stress induced depolarization of mitochondrial membrane, resulting in release of cytochrome *c*, followed by the activation of caspase pathway, thus leading to apoptosis [55–56]. Limited work was done concerning the genotoxic effect of FPN. FPN was found to be genotoxic and mutagenic to rats and induced high rate of chromosomal aberrations and micronuclei [57]. FPN entering the mice brain within a few minutes and locally converted into the corresponding sulphone as the main metabolite for FPN within 2–4 h [58]. As reported by pervious study, exposure to FPN mediates apoptosis through the oxidative stress-related pathway [59]. These results were confirmed by Badgujar et al. [60] who reported that FPN afforded neuronal cell death. Recently, it was reported that FPN induced an increase in ROS generation in association with reduction in the mitochondrial membrane potential [61]. FPN significantly augmented the release of cytochrome *c* and the mitochondrial translocation of Bax, enhanced the activity of cleaved caspase-3, caspase-9, and markedly down-regulated the expression of Bcl-2 [62]. These findings indicated that FPN triggers intrinsic apoptosis via the mitochondrial signaling pathway that is initiated by the generation of ROS [56,58].The mechanism of FPN toxicity involved its effect on mitochondrial bioenergetics and an alteration in calcium homeostasis, which led to a decrease in ATP synthesis with consequent cell death by necrosis [30]. Looking for potentially neuroprotective agents from natural products could attenuate oxidative stress-induced neurotoxicity might be helpful in the prevention and treatment of neurodegenerative disorders [63]. From the obtained results, it was evident that neuronal cell death due to oxidative stress induced by FPN intoxication was significantly suppressed by TLE treatment through significant reduction in mRNA expression for both apoptotic genes (caspase-3 and Bax) in brain tissue (Figure 1). Gallic acid detected in methanolic extract of TL had the potency to down-regulate the protein expression and activity of caspase-3, an essential effectors molecule in the course of programmed cell death [64]. Ellagic acid had the potency to reduce apoptosis and neuro-inflammation and could be used as suitable therapeutic agent for moderate brain damage in neurodegenerative diseases [65]. Neuroprotective effect of *Terminalia* species extract was probably achieved due to its phytochemical antioxidant constituents, such as flavonoids (e.g. polyphenols), saponins (triterpenoid ivorenosides A, B, and C), tannins and ellegic [47,66]. Many of these constituents have been shown to readily cross the blood–brain barrier to exert central nervous system activities, which includes anti-neuroinflammatory, neuroprotective, chemoprevention, antioxidant activity [67]. Gallic acid and ellagic acids are previously reported to inhibit cytochrome p450 enzyme and protect against mitochondrial dysfunction [68]. Tannins act as radical scavengers and also play a role in treating various degenerative diseases [69]. Since the extract is presumably a mixture of several compounds, it may be possible that some compounds are responsible for antioxidant activity and others are responsible for the anti-apoptotic effect against FPN-induced neurotoxicity. The change in oxidative stress and expression level of apoptotic genes in brain tissue in rats exposed to the FPN corroborated the histopathological lesions observed in the present study. These observations indicated marked changes in the overall histoarchitecture of the brain in response to FPN. These changes could be due to FPN toxic effects primarily by the generation of ROS causing damage to the various membrane components of the cell. Administration of TLE to FPN-exposed rats showed improvement of histopathological alteration in comparison with FPN intoxicated group.

## Conclusion

The findings of the present study support that the 80% TLE is considerably an effective radical scavenger due to the presence of flavonoid and phenolic compounds that possess a neuroprotective (antioxidant) effect and showed an activity against FPN-induced neurotoxicity. These findings provide valuable scientific support for traditional claims in the management of FPN toxicity.

## Availability of data and material

There are no restrictions to the availability of any materials and data upon request.

## Abbreviations

CAT: catalase
FPN: Fipronil
GSH: reduced glutathione
LPO: lipid peroxidation
MDA: malondialdehyde
SOD: superoxide dismutase
TL: *Terminalia laxiflora*
TLE: *T. laxiflora* methanol extract
